# Changing the culture of assessment: the dominance of the summative assessment paradigm

**DOI:** 10.1186/s12909-017-0912-5

**Published:** 2017-04-28

**Authors:** Christopher J. Harrison, Karen D. Könings, Lambert W. T. Schuwirth, Valerie Wass, Cees P. M. van der Vleuten

**Affiliations:** 1 Keele University School of Medicine, Keele, Staffordshire ST5 5BG UK; 2 Faculty of Health, Medicine and Life Sciences, Maastricht University of Maastricht, Maastricht, The Netherlands; 3 Flinders Medical School, Adelaide, Australia

**Keywords:** Feedback, Summative assessment, Programmatic assessment

## Abstract

**Background:**

Despite growing evidence of the benefits of including assessment for learning strategies within programmes of assessment, practical implementation of these approaches is often problematical. Organisational culture change is often hindered by personal and collective beliefs which encourage adherence to the existing organisational paradigm. We aimed to explore how these beliefs influenced proposals to redesign a summative assessment culture in order to improve students’ use of assessment-related feedback.

**Methods:**

Using the principles of participatory design, a mixed group comprising medical students, clinical teachers and senior faculty members was challenged to develop radical solutions to improve the use of post-assessment feedback. Follow-up interviews were conducted with individual members of the group to explore their personal beliefs about the proposed redesign. Data were analysed using a socio-cultural lens.

**Results:**

Proposed changes were dominated by a shared belief in the primacy of the summative assessment paradigm, which prevented radical redesign solutions from being accepted by group members. Participants’ prior assessment experiences strongly influenced proposals for change. As participants had largely only experienced a summative assessment culture, they found it difficult to conceptualise radical change in the assessment culture. Although all group members participated, students were less successful at persuading the group to adopt their ideas. Faculty members and clinical teachers often used indirect techniques to close down discussions. The strength of individual beliefs became more apparent in the follow-up interviews.

**Conclusions:**

Naïve epistemologies and prior personal experiences were influential in the assessment redesign but were usually not expressed explicitly in a group setting, perhaps because of cultural conventions of politeness. In order to successfully implement a change in assessment culture, firmly-held intuitive beliefs about summative assessment will need to be clearly understood as a first step.

## Background

Over the last few years, there have been calls within the general educational establishment to move away from the dominance of high-stakes testing at the end of a period of learning (usually referred to as ‘assessment of learning’) towards a focus on multiple low-stakes assessments throughout the period of learning, combined with rich feedback (usually referred to as ‘assessment for learning’) [1]. Within medical education, there has been a developing interest in programmatic assessment, which seeks to incorporate both assessment for learning and assessment of learning [2, 3]. Although in some cases this appears to have been implemented successfully, the introduction of this novel approach has been more problematical in other institutions [4–8].

Although designers of assessment programmes may be attracted by the theoretical concepts of programmatic assessment, they may be hesitant to proceed with a radical change of assessment culture because of the reported difficulties in implementation. They will also recall that, within medical education, implementation of previous innovations has not always been straightforward. For example, although constructivist approaches to learning, such as problem-based learning (PBL), have gradually become more popular over the last 30 years, the change has often been controversial and not universally accepted by clinical teachers in particular [9, 10]. Enthusiasts for assessment culture change therefore need to consider the evidence about which factors underpin, or hinder, successful culture change in other fields.

The challenges involved in modifying an organisation’s culture are immense, as organisations are typically inherently resistant to radical change. According to Johnson [11], who applies a socio-cultural perspective to organisational change, one of the reasons for this inertia is because the managers responsible for change share common core underlying beliefs and assumptions about the organisational culture. Johnson defines this as a *paradigm*. The assumptions are typically implicit as they are the organisation’s values which are taken for granted. Any potential change tends to be viewed through the filter of the paradigm. This paradigm of beliefs is itself part of a wider ‘cultural web’ of an organisation. Johnson [11, 12] defines a number of elements which make up the cultural web. The *power structures* of an organisation are often closely linked to the paradigm, as the most powerful members of the organisation are likely to be most closely associated with the core assumptions. The formal and informal *organisational structures* tend to reflect power structures and ensure that certain relationships or structures are particularly valued within the organisation. Organisations typically adopt a number of *rituals and routines* that members follow, often without thinking. *Stories* are often relayed down through an organisation, recalling significant events and people from the organisation’s history. These serve as demonstrations of what the organisation values. Similarly, *symbols* can provide important insights into an organisation’s values; at their simplest, symbols may be logos, but they can also be indicators of power, such as reserved car-parking spaces. Organisations also have formal *control systems* which monitor progress in certain aspects and therefore ensure where attention is focussed. Overall the interaction between the paradigm and the wider cultural web leads to an assumption that “this is the way things are done around here” [11]. If managers are faced with pressure to change, they will typically redesign in a way which is consistent with the prevailing paradigm and the wider cultural web of the organisation. So a change in assessment tool, say from multiple-choice questions to short-answer questions, may be relatively easy to implement as this change does not challenge the underlying paradigm. However, a change from an assessment of learning culture to one based on assessment for learning is much more fundamental. Johnson [11] argues that the cultural web needs to be made explicit before more radical change can be contemplated.

Once an organisational culture has been understood, successful change also depends on other factors [13]. In particular, the consequences for the individual must be considered carefully. It is not possible to change an individual’s behaviour simply by changing the culture [13]. Instead, a climate of ‘psychological safety’ needs to be created in order to allow individuals to feel personally involved. They need to be able to evaluate for themselves the beliefs and values inherent in the new culture, and to examine the consequences for themselves as an individual [13].

Educationalists have sometimes tended to act as if they were unaware of this evidence from the change management literature, even though it is likely to be equally challenging to implement change of an educational culture or learning environment as it is to change the culture of a business [14]. The importance of involving students as partners within instructional redesign is often overlooked despite evidence that incorporating students’ perceptions can improve redesign [15]. Instead, students’ input is frequently limited to evaluations about the quality of teachers. Failure to incorporate students’ perceptions when redesigning a learning environment leads to feelings of alienation and disempowerment among students, with resultant adverse effects on motivation [16]. In a previous study on the implementation of programmatic assessment, it was clear that assessments designed to be formative were perceived by the learners to be summative, which resulted in reluctance for the students to take part in the assessments [7]. These perceptions had not been explored at the implementation planning stage.

Teachers are also important stakeholders to be considered when designing instructional innovations, yet they are often not fully involved in the design process, even though they are expected to enact the redesigned learning environment in practice [17]. A lack of interaction between designers and teachers can leave the latter group unsupported while expected to implement educational innovations in contexts which may be less than ideal [17]. This was evident in the previously-mentioned study of programmatic assessment implementation. Failure to fully involve the clinical supervisors at the design stage left them feeling frustrated as they felt the new system did not value their role as judges [7]. Top-down implementation of new assessment practices has failed in the past to bring about a desired change in the behaviour of teachers or students [18].

To aid a much closer collaboration between students, teachers and instructional designers, the Combination-Of-Perspectives (COOP) model has been proposed as a way of visualising the different stakeholders involved [19]. This process of incorporating multiple stakeholders’ perceptions when (re)designing a learning environment is usually referred to as participatory design [20]. Participatory design does not imply that students should have full control over the design process as they do not necessarily have the expertise to design an environment which is optimal for their learning. Instead it is proposed that they should be partners in a design process that also uses the expertise of teachers and instructional designers. Within the general educational literature, some successes have been reported for the use of a participatory design approach in an undergraduate setting [21].

It would, however, be simplistic to assume that change can proceed successfully once stakeholders’ perceptions are acknowledged. One reason why change is so difficult is that many of the stakeholders’ perceptions are much more than mere ideas; instead they represent their own personal epistemologies [22]. These are intuitive but firmly-held beliefs which we all hold about the world around us. Any clinician who has tried to convince a patient to adopt a healthier lifestyle will recognise the strength, and often fixed nature, of personal epistemologies, which are frequently in direct conflict with strong medical evidence [23]. Similarly, clinicians themselves often fail to incorporate evidence from research trials into their daily practice because the evidence does not sit comfortably with their underlying beliefs [24]. This failure to incorporate evidence can also be applied to assessment in medical education. All students and teachers will have experienced assessment in various forms, both within the school education system and since entering medical school. As a result, they are likely to have formed their own beliefs about how an assessment should be. It may not be easy to change these belief systems, even if they are in conflict with evidence-based research which demonstrates how a modern assessment programme should be designed. The previous studies about receptivity to feedback in different assessment cultures provided some evidence about students’ epistemologies regarding current assessment and feedback practices, as well as their perceptions about their teachers’ beliefs about these matters [25, 26].

If a medical school is seeking to consider a change of assessment culture towards one in which ‘assessment for learning’ is promoted, a number of factors will therefore need to be considered. Clearly it would be vital to engage all stakeholders, who in this case comprise students, teachers, assessors and senior members of faculty. The institution should seek to understand the shared common beliefs which underpin the assessment culture. In addition, the consequences of the change for individual stakeholders need to be considered by exploring their beliefs about the proposed change. The need to address all these issues together helps to explain how difficult it is to bring about organisational culture change.

Given the difficulty in bringing about organisational culture change, incremental change is often preferred [11]. There are a number of steps which would need to occur to convert an assessment programme from one based on ‘assessment of learning’ to one which incorporates ‘assessment for learning’ principles. A key initial step would be to ensure that students make more use of post-assessment feedback. Attempts have been made to combine high-stakes assessments with feedback. Harrison et al. delivered feedback via a website to all students following a summative objective structured clinical examination (OSCE) [27]. Although almost all students viewed the website, there was considerable variation in how intensively they viewed the feedback. In particular, students who had only just passed the OSCE made least use of the website compared with those who had performed well, or those that had failed the assessment. Further work demonstrated that summative assessments created a powerful culture which was dominated by students’ fear of failure and subsequent punishment [25]. Interactions with teachers, peers and others reinforced the need to avoid failure and paradoxically focussed more attention on students who failed rather than on those who had passed the assessments. Furthermore, the feedback provided after the summative assessment was not regarded as relevant for future learning in the clinical workplace, but was only seen as relevant for future summative assessments. In a more recent study, students made more use of feedback after a progress test when the test was integrated into a comprehensive program of assessment, which included mentoring with scaffolding of feedback, than when it was used in a summative manner [28].

We therefore wanted to explore an institution’s readiness to adopt initial changes which would help an organisation move towards an assessment for learning culture. We were interested in the design solutions which would be proposed by stakeholders, in the factors which would support the implementation of this change and the barriers which would be faced, and the influence of stakeholders’ epistemological beliefs about assessment on any proposed redesign. This led to the specific research questions:

1. When stakeholders are asked to redesign a summative assessment culture to ensure that students make more use of post-assessment feedback, what solutions do they suggest?

2. How do the stakeholders’ personal and collective epistemological beliefs about assessment influence their redesign of the assessment culture?

## Method

### Context

The study took place at Keele University School of Medicine, which has approximately 130 students per year. It is one of the newest British medical schools, graduating its first cohort of doctors in 2012. Progression through, and exit from, the course relied on a series of summative assessments. There is a focus on the provision of feedback to all students following high-stakes assessments, although there is no obligation for students to make use of the feedback. The school has a tradition of respecting the student voice in a number of ways. Student representatives serve on all the major school committees (including for assessments). They also provide feedback about the course on a regular basis, via online surveys and face-to-face interactive group meetings with faculty members. At these meetings, students learn what has changed in the course as a result of their feedback. They also make suggestions for further changes.

Feedback is provided following both written assessments and Objective Structured Clinical Examinations (OSCEs). Written assessments comprise a mix of single-best-answer and short-answer format questions. These are aligned to the particular year’s learning objectives; there is no longitudinal progress test. For the single-best-answer questions, students receive feedback around their performance in different specialty areas (e.g. respiratory medicine), regarding the specific clinical presentation (e.g. cough) and the focus of the question (e.g. choice of investigation). This information is available on a website. Students can view how their performance compares with the cohort as a whole. For students who achieve a satisfactory mark in the short-answer questions, small group sessions take place to enable students to view their marked papers alongside the ‘model’ answers. Students with an unsatisfactory performance receive one-to-one feedback. OSCE examiners provide feedback in two ways in Years 3 to 5 of the programme. Firstly, the breakdown of marks for each station, and for each domain across stations, is provided on a website. More details of this have been published elsewhere [27]. Secondly, examiners provide verbal feedback of up to 90 s by speaking into an iPad once the student has left the room. This is recorded by an app and is uploaded into the feedback website, where it is available for the student to listen to. More details of this have been published elsewhere [29].

### Participatory redesign meeting

We conducted a small-group meeting based on the principles of participatory design. Participants were purposively sampled to recruit five medical students (from Years 3 to 5 of the programme), two clinical teachers (with responsibility for Years 3 to 5) who are also experienced OSCE assessors and two senior members of the faculty, who have responsibility for overall curriculum design (and who were known to already have an awareness of the broad concepts of ‘assessment for learning’). By aiming to have more than half the participants as students, it was hoped to minimise hierarchical power issues within the group. Potential students were approached by members of the School’s academic and administrative team (not involved in the research) solely on the basis of their likely confidence to cope in a meeting with senior members of faculty. Previous involvement in faculty structures or curriculum development was not considered in the recruitment process.

A joint meeting was held to include all participants. The lead researcher provided a short presentation explaining why the educational research evidence would support a change in assessment processes to ensure that feedback was used, along with evidence demonstrating the difficulties in ensuring that students make use of the feedback. The problem with feedback following OSCEs was used as a concrete example, but the participants were encouraged to consider the wider problem of feedback following assessments as a whole. They were challenged to use blue sky thinking to design radical solutions for this problem. Participants spent some time alone writing their own ideas on Post-it notes. They then formed pairs of a clinician or faculty member and student (or in one case a trio comprising two students and a clinician) to share ideas and develop agreed priorities. Participants then formed mixed groups of 4 or 5 to further develop ideas and priorities for change. The group then reformed as a whole to compare proposed solutions and discuss them in more detail. Once they had come up with their suggestions, the group members were then encouraged to critically challenge their proposals in order to explore the underlying factors which would support (or hinder) successful practical implementation of the assessment change. The meeting lasted approximately two and a half hours.

### Individual follow-up interviews

A potential risk of group meetings is that respondents may provide socially desirable responses, while the strength of their own personal beliefs may not be sufficiently clear. When sharing a room with senior faculty, students and potentially even clinical teachers may not feel sufficiently empowered to voice their concerns about how the change will affect them. As a key element of successful change management requires an understanding of the consequences for individual stakeholders, it was felt important to ensure their beliefs were captured accurately.

Each group participant was therefore invited to an individual follow-up meeting in the following weeks. Seven participants were interviewed within 1 week of the participatory redesign meeting. For logistical reasons, one faculty member was interviewed 8 weeks later and one student was unavailable. At these interviews, participants were reminded of the suggested solutions which the group proposed. Using a minimally-structured interview process, participants were encouraged to consider the personal consequences for them as an individual, if the proposed changes were to be implemented. This approach was designed to allow further exploration of stakeholders’ beliefs towards assessment and feedback. The interviews each lasted approximately thirty minutes.

### Data collection

The post-its completed by individuals and pairs were collected and transcribed. Those completed by individuals were labelled as being written by students or clinicians (to incorporate both faculty members and clinical teachers). This was done to achieve sufficient anonymization. The outputs from the sub-groups were written on flip charts to facilitate sharing with the whole group. These were also transcribed. The participatory redesign meeting as a whole, and the individual meetings, were audio-recorded and transcribed. These were supplemented by field notes collected by an experienced observer, who had a specific remit to observe interactions between group members.

### Data analysis and interpretation

Data were analysed from a sociocultural perspective, using Johnson’s cultural web as a lens [11], in order to understand aspects of the organisational culture as well as individual beliefs. Although the proposed changes to the assessment culture were interesting in themselves, the interaction between participants, and in particular the relative influence of students in moulding the proposals, was of specific interest. The transcripts and field notes from the group meeting were analysed carefully to understand how the proposed changes were developed (or blocked) within the group. We aimed to identify collective and personal epistemologies held by the group as a whole and by individual members.

Coding of all data was performed by the lead researcher. In order to validate emerging concepts, the participatory design meeting transcript was also thoroughly read by a second researcher, while the individual interview transcripts were analysed by a third researcher. Regular videoconference discussions with the wider research team helped modify the coding and the conceptual analysis arising from it. In recognition that our analysis framework assumes that data are co-constructed by interactions between researchers and participants, we provide the following contextual information: CH, LS, VW are medical doctors with a major involvement in medical education research and development; CvdV, KK have backgrounds in psychology. All members have a strong interest in the effects of assessment on learning. The lead researcher knew well the two faculty members who participated and had previously briefly met the clinician participants. He had only met one of the students prior to the group meeting.

Ethical approval was obtained from Keele University School of Medicine Ethics Committee.

## Results

### Proposals to enable greater use of post-assessment feedback

A summary of the suggestions which emerged from the different phases of the redesign meeting is shown in Table 1. Three principal ideas were discussed in detail in the whole group: the need for more authentic assessment, the potential to give feedback without (or before) the issuing of grades and the role of one-to-one mentoring to support the interpretation of the feedback.

**Table 1:** Development of main Ideas through participatory redesign phase and individual interviews

		Need for more authentic assessment	Provision of feedback without (or before) grades	Provision of one-to-one mentoring following assessments	Improved quality of feedback	Feedback regarding correct ‘model’ answers	Improved multi-source feedback by peers	Other
Individual post-it suggestions	Students	0	1	2	3	4	0	6
	Clinicians	1	0	5	3	1	0	3
Pairs/trios		1	1	4	2	0	0	0
Sub-groups		1	1	2	2	0	0	0
Whole group		Discussed in detail. Largely supported in the group.	Discussed in some detail. Discussion led by two students. Clinicians implicitly critical.	Discussed in detail, but largely dismissed as logistically impractical.	Discussed very briefly – difficult to provide feedback when considering mark of borderline candidates.	Not discussed.	Brought up briefly by clinician near the end of the meeting	Not discussed
Individual follow-up interviews	Students	All discuss need for greater authenticity	One students remains strong advocate, others critical.	All supportive, while recognising practical constraints	Not discussed		Mixed views	
	Clinicians	¾ discuss need for greater authenticity	All are critical	One clinician supportive, others critical	Not discussed		Mixed views	

Numbers refer to the number of individuals or groups who raised a particular topic. Note that individuals completed more than one post-it per person

#### Authenticity

There was broad agreement that current assessments, especially OSCEs, were often inauthentic and failed to detect the attributes that will be needed for a medical career:

*We still go for the sort of high stakes, set piece drama and it does feel like a drama; whether it’s the theatricality of the OSCE or the crescendo of stress prior to the written exam. So it’s a drama and there’s a little bit of value in that in terms of testing resilience and ability to cope with stress but on the whole, stress that we want graduates to be able to cope with is more sustained. Faculty member 2, individual interview.*

There was widespread resentment from students that engagement with clinical workplace learning failed to be reflected in assessment performances:

*I think definitely you really do notice the people who don’t go to wards and then you talk to them after exams and they did better in the OSCEs and you think, ‘How did that work? I slaved all year.’ Student 3, group meeting.*

The perception was that students were able to do last-minute superficial learning which was rewarded by the assessment processes but which failed to equip them with skills for ongoing workplace learning:

*They did two weeks of really good OSCE revision, nailed the OSCEs, come back in three weeks’ time and they don’t know what’s going on. Student 5, group meeting.*

Time constraints left students frustrated that they were unable to adequately demonstrate their abilities:

*I don’t think you ever quite leave an OSCE with the time constraint thinking you’ve really flourished in that exam and you’ve really shown them, ‘This is everything I know on this. If I’ve forgotten anything then it’s fine,’ because you’re always time constrained. So you’re cutting out things in your own head, trying to leave things out just to get it into that little tiny window. Student 4, group meeting.*

Restrictions on time also frustrated examiners’ efforts to differentiate between students:

*One of the things that I see a lot of in OSCE stations is that technique is wonderful but they’re just doing this stuff in the correct order as they’ve been taught but not being able to actually produce what the abnormalities are and so they can’t then make a proper working diagnosis. Those students who I know go to the wards a lot are able to often come out with the correct diagnosis but we don’t have time to tease that out. Clinical teacher 2, group meeting.*

The scenarios that could be assessed in an OSCE format failed to reflect the realities of the clinical workplace; attempts to address this were clumsy:

*In OSCE stations it tends to be you on your own whereas most of the work I’ve ever done is being part of a team. And that doesn’t seem to be assessed very widely in OSCE stations, the team working. Clinical teacher 2, group meeting.*

The lack of authenticity led to feedback from OSCEs being discounted:

*When you get feedback from a short OSCE you’re also thinking in the back of your mind, ‘Well, if they would have seen me do it on the ward,’ or, ‘I’ve done 40 cannulas but they’ve only seen me do that one and I couldn’t get it into the vein on that funny arm but I can do it on the endoscopy list.’ Student 5, group meeting.*

However, there was no consensus about how to make the assessments more authentic. Attempts to redesign were typically minor rather than radical:

*Do you think OSCEs could be better designed within the sort of constraints that we have to pick out people who are good clinically? Faculty Member 1, group meeting.*

Suggestions considered included lengthening OSCE stations, a return to long cases at the end of each clinical attachments, or a desire to make workplace-based assessments more rigorous and ‘objective’. Increasing the time for OSCE stations was thought to support better integration of knowledge and skills assessment.

*If you had more time as well it would allow more questioning which would allow the examiner to probe more about why you’re doing certain things. Student 1, group meeting.*

It was also felt likely to support pre-assessment workplace-based learning:

*You’d have to be pretty gutsy to work up to a half-hour exam not having been on the wards for a while before that. Student 5, group meeting.*

However, there was an unresolved tension between the need for greater authenticity and the belief in the need to maintain or enhance standardisation and reliability to ensure fairness:

*I guess the long cases are much harder to standardise aren’t they, I presume that’s why we’ve moved away from them and you can’t so easily do a number of long cases so that you kind of get that spread and you get a truer picture. Whereas you can do lots of OSCE stations. But what they’re actually going to be doing when they hit the wards is see people for the first time, taking a full history, doing a full examination, trying to come up with something as a result of it aren’t they. So in that sense a long case is more reflective of what they’re going to do, they’re not going to be asked to just go in and do one tiny little bit of somebody for eight minutes. So I could see the point of that but I just, you’d have to work really hard at how you did that in a way that was fair and equitable. Clinical teacher 1, individual interview.*

Current attempts to square this circle were not seen as successful:

*Some of the [workplace-based] assessments you get really aren’t up to it - they’re not standardised enough. There’s too many variables. And I think they should be more formalised. Clinical teacher 2, group meeting.*

There were also apparent dangers in abandoning current assessment formats as they were seen as helpful preparation for examinations later in the learners’ careers:

*That sort of OSCE format then goes on into future postgraduate exams and I think because it is such a stressful format I think there are really quite big advantages to having had quite a bit of experience of it so you’re not just thrown into it when you’ve paid a large amount of money to do this exam and a lot rests on it. Clinical teacher 1, individual interview.*

#### Feedback without grades

A single student’s suggestion of providing feedback without grades generated significant group discussion. It was advocated as a way of enhancing intrinsic motivation:

*The issue is trying to get students more intrinsically motivated rather than just, ‘I passed and am competent,’ because that’s why I would assume the excellent students are looking over their feedback more than the students that have just passed because they are wanting to better themselves. It takes the competency out of the equation and pushes people to progress their performance each time they come to it. Student 1, group meeting.*

However, many felt that this would result in them converting the narrative feedback into a numerical mark, in order to determine whether or not they had passed the assessment, with adverse effects on their receptivity to feedback:

*You’d just be preoccupied with going, ‘Have I done enough to pass this, then?’ rather than actually taking on board the feedback. Student 2, group meeting.*

This approach could be potentially misleading as there were mismatches between the narrative feedback and numerical marks:

*In our group students were saying that with the audio feedback they’re currently getting from the OSCE stations what they tend to find is that the stations that they’ve done well in there’s usually quite a lot of feedback on what they could do to improve it whereas the stations that they’ve just passed there tends to be quite encouraging feedback and not so much of the what they could do better. So I don’t know how well you can link it to the grade, really. Clinical teacher 1, group meeting.*

The mismatch was exacerbated by a focus on the pass-fail mark, especially for those students whose performance was only just satisfactory. Examiners needed to focus primarily on the mark, which was seen as the more important issue, with feedback of secondary priority.

*I give a lot of feedback, probably too much feedback, to the ones at the brilliant end with all the things they can do better because you don’t have to fret about it. The ones who are borderline, you’re fretting about them and then you’ve only got a few seconds to give the feedback and so it messes up the feedback to those who particularly need it. Clinical teacher 2, group meeting.*

Failure to award grades was seen as incompatible with one of the fundamental purposes of assessments:

*Obviously at some point there has to be a decision of whether you’re fit to practise or not so there has to be some sort of a grade at least at the end. Clinical teacher 1, individual interview.*

Grades provided clarity and reassurance:

*I think they give you some security in knowing that you’re doing well and where you’re at. And I think grades are a lot easier to process in student’s mind, say if it was A, B, C or whatever. If you were looking at a C grade you know you’d need to probably up your game whereas if you’re at an A, what you’re doing is right. Student 4, individual interview.*

More fundamentally, there was a lack of belief in the evidence underpinning the provision of feedback without grades:

*This is a difficult one because I know what I’m meant to think about it, because I know that there’s reasonably good evidence that feedback is more effective without the grades but I can’t quite believe it. Faculty member 2, individual interview.*

#### Mentoring

There was much discussion about the theoretical benefit of some form of mentoring. The discussion concentrated mostly on one-to-one post-exam feedback (how to do better in future exams) rather than long-term supportive coaching. Mentoring was seen as a way of encouraging students to engage meaningfully with the feedback.

*You all get bad feedback sometimes and it can be a bit of, it’s hard to see the wood for the trees kind of thing if you’ve got quite a lot of things that you need to kind of like improve upon and I think talking to someone can first of all make you think about it more rationally, because if you get bad feedback it can just seem a bit oppressive. Student 1, individual interview.*

Many barriers to successful implementation were identified. The timing of any mentoring needed to be soon after receiving post-assessment feedback, but also needed to be long enough to be meaningful. Students recognised that clinical service commitments meant this would be unlikely to be achievable.

*I think that’s [mentoring] probably the least practical thing that we discussed. Probably one of the most beneficial, though, because it would be amazing to do it. But I think that’s a very tough thing to do, especially getting specialists to give you feedback on that area because everyone’s got so much work on their plate as it is. Student 4, individual interview.*

A suitable mentor was seen as someone who was familiar with the requirements for the assessments, not necessarily one with an ongoing relationship with the student.

*No they don’t have to know us at all they just have to know the exam system and be rounded enough to be familiar with all areas of the exam possibly. Student 3, individual interview.*

A tension existed between having a specialist who could give credible feedback in their own area versus a tutor who could help more broadly with all aspects of assessment. On the one hand feedback from a specialist was seen as more valuable:

*I think the student would respect the feedback more that it’s come from a cardiologist because I was rubbish at cardiac exam rather than it coming from some other specialty where you go, ‘How many have you done? You probably haven’t done one of those since med school.’ Student 4, individual interview.*

In contrast, feedback from specialists could impair preparation for assessments:

*If you’ve got a consultant in that aspect, they’re going to start throwing extra stuff into that exam that you don’t need in an OSCE. So you can almost be, on the flipside, disadvantaged by having a super specialist who’s like, ‘Oh make sure you check for this, you’ve done this, you’ve done that,’ because they don’t know what you need in an OSCE. Student 2, group meeting.*

The challenges involved in finding enough mentors to spend enough time with students needed a huge change in organisational culture:

*I think in terms of overcoming it, it would mean a fundamental shift in the culture of a medical school faculty and in the priorities that faculty had. Faculty member 2, group meeting.*

While there was recognition that such a change would be popular, it was not thought to be worth the culture change if the main aim was to improve assessment performance rather than clinical practice:

*If it’s just so that you can do better in exams I think that has to be questioned. As a student I’d want that but, ‘Is that the best way to use resources?’ But if actually you perform better in clinical practice as a result of that feedback then the argument for me would be compelling. I’ve no idea how we could judge that. Faculty member 1, group meeting.*

Compromises considered making the mentoring available on a voluntary basis or targeting certain year groups:

*Ideally obviously everyone would have it, it would be compulsory but I think as a compromise even just making some appointments available and seeing the popularity and engaging in it because at the moment obviously you’re only getting those appointments if you’re failing. Student 2, individual interview.*

*Having thought about it a bit, the logistics of doing it for all five years, I think, would make it very hard but we may be able to identify key points in the five years, where we can deploy the people who would be good at it. It may be that that each student has this sort of experience twice during their five years. Faculty member 2, individual interview.*

The consensus was that, while popular, this change could not easily be practically implemented:

*The conclusions of everybody having a one to one, making sure everybody engages with it would be amazing. And just briefly talking to my housemates about it they’re like, yeah that would be so good because I think there’d be a lot of support for it but the blue sky thinking isn’t reality. Student 2, individual interview.*

### The influence of personal and collective beliefs on assessment redesign

Within both the group discussion and the individual interviews, the summative paradigm was a dominant factor. Participants’ prior experiences of assessment influenced redesign proposals. Within the group, there was evidence of a hierarchy with senior members exerting more influence.

#### The summative paradigm was dominant

Most of the beliefs expressed were firmly rooted within the summative assessment paradigm. Discussions were dominated by the need to get through assessment hurdles, rather than becoming a good doctor. The primary focus of assessment was the pass-fail mark and the need to prevent unsafe students qualifying as doctors:

*So the good and excellent, that’s almost an irrelevance. It’s the ones that are no good that we’re trying to find, surely. Clinical teacher 2, group meeting.*

The paradigm included a belief in the need for numbers/grades as a form of perceived objectivity or rigour. There were negative attitudes to changes to assessment models if this rigour and objectivity were to be lost. Within this paradigm, the aim of the feedback was principally that it should help the student do better in future assessments, or allow more chance to check on the accuracy of the marking.

*We should have a one-to-one meeting pretty much after the exam and one before the next assessment to see, ‘What’s the action plan to do well and where can you improve from last time?’ Student 5, group meeting.*

There was a sense that for some, this paradigm worked for them and the majority of students, so they were reluctant to change it radically.

#### Influence of prior assessment experience

Prior experience of assessment and feedback, whether positive or negative, acted as another filter through which proposed changes were viewed. Participants frequently recited stories of their own personal assessments. As these typically occurred within a summative assessment framework, they acted as a further reinforcement of the summative paradigm. For example, discussion about the need for greater authenticity in assessments reverted to the need for standardisation:

*When we had real patients this year I think some people get different findings you think, ‘Oh Christ. Was it all the same? Student 4, group discussion.*

However, prior experience of a different assessment culture supported desire for change. While most participants felt mentoring was impractical, one member was a strong advocate because of the impact on his own career:

*From my own personal experience, when I was at Medical School, I was sadly ignored for several years and then an inspirational mentor came along, and then I grasped what it was all about and it drove me to improvement. Clinical teacher 2, individual interview.*

#### Power was persuasive within the group

Although superficially there was good interaction between group members, with students contributing frequently and in detail, they appeared less able to influence the group’s opinion as a whole. In contrast, a single clinician’s suggestion about the need for greater authenticity appeared to ‘strike a chord’ with most of the rest of the group. In the end, virtually every member seemed broadly in agreement that assessments are often inauthentic, with students particularly vocal in support.

Within the group setting, a variety of techniques were deployed by participants to encourage or discourage discussion of proposed changes to the assessment culture. Students commonly provided explicit support to other students expressing ideas, but this behaviour was rarely used by clinicians, who tended to express more implicit support. When disagreeing with other group members, students tended to disagree explicitly with other students but rarely with clinicians. On the other hand clinicians and faculty members rarely expressed explicit disagreement, especially towards students, preferring a more implicit approach instead, often combined with a questioning style or “sitting on the fence”:

*It sounds almost as if the grade gets in the way of learning from the feedback either because of the phenomenon of just passing, so knowledge of the grade changes it, but also somebody like me if I got the feedback without the grade I’d be trying to calculate my grade and so wouldn’t necessarily engage. So does the grade get in the way? Faculty member 2, group discussion.*

The clinicians and faculty members were more explicit in expressing their beliefs in the individual interviews.

*I can’t imagine getting beyond trying to translate the feedback into a grade. My positive responses in terms of learning would be stymied by my internal algorithms trying to work out whether these words mean I’ve passed or failed. Faculty member 2, group discussion.*

Students who remained silent in the group when certain ideas were discussed were much more forthright in expressing their opinion in the follow-up interviews:

*Not giving the grades first … I think mentally that would drive you insane. Student 3, individual interview.*

Clinicians used a couple of techniques to control the flow of discussion within the group. Firstly, they sometimes abruptly changed the topic being discussed. This was usually successful in ending the group’s discussion of a proposed change. Another technique employed was to ask the group a focussed question, which was also effective in moving the group discussion in a different direction. For example, in the middle of a discussion on making assessments more authentic, a clinical teacher said:

*Can I ask a question? Do you think that you have too few, the right amount or too many assessments as you’re going through your medical school? Clinical teacher 2, group discussion.*

While the expression of individual beliefs was much clearer in the follow-up interviews, there was little evidence that the beliefs had been changed by what they had heard in the group setting.

## Discussion

We aimed to explore how a mixed group of stakeholders would redesign a summative assessment culture to ensure that students would make use of post-assessment feedback. In particular, we were interested in how participants’ personal and collective beliefs about assessment influenced the redesign. We found that participants shared common assumptions and beliefs about the importance of the summative assessment paradigm. Discussion about the redesign focussed on the use of feedback to help students pass future assessments, rather than using the feedback to help students become better doctors. Elements of an ‘assessment for learning’ culture, such as long-term mentoring and the provision of feedback without grades, were considered but not seen as practical ideas for implementation. Participants relied heavily on their own prior assessment and learning experiences to guide their views on what changes were possible or desirable. Although discussion between participants from different backgrounds demonstrated good participation in the group discussion, disagreement was often voiced in an implicit manner and senior clinicians and faculty members appeared to exert more influence than students. Follow up interviews demonstrated that underlying personal beliefs were largely unchanged by the group discussion. There appeared to be a shared common assumption that (to paraphrase Johnson [11]) “summative assessment is the way things are done around here”.

The dominance of the summative assessment paradigm, and the desire to make small changes without disrupting the paradigm, is consistent with Johnson’s work on the conceptual challenges organisations face when confronted with evidence of the need to change [11]. Johnson argues that an organisation’s strategy is based on common, often unspoken, assumptions which are shared by members of the organisation. As a result, elements of an organisation’s culture are ‘taken for granted’. In our study, participants appeared to take for granted that summative assessment is the way in which students should be assessed. Suggestions for change were therefore predominantly aligned with the paradigm of summative assessment.

Despite contributions from all members, it is unsurprising that the senior clinicians and faculty members appeared more capable of influencing the outcome of the discussion. Members of a medical team formed on an ad hoc basis typically have preconceptions regarding the distribution of power, based on prior experiences or stereotypes [30]. The implicit ways in which participants disagreed with each other in the group has similarities with Brown and Levinson’s theory of politeness, as described in the field of linguistic pragmatics [31]. In their theory, they describe how speakers employ strategies to avoid causing offence while still trying to communicate what they desire. Examples include the use of indirect language, statements of general rules or questions. Recently, Ginsburg and colleagues [32] have applied this theory to the written feedback provided by faculty to learners; they found the use of non-literal language was common in these circumstances and enabled faculty members to “save face”. Politeness in interactions inevitably causes confusion and can even be dangerously unhelpful in certain situations [33]. It was clear from our study that politely-expressed comments, or even silence, in the group did not adequately represent more firmly-held personal beliefs which became evident in the individual interviews.

### Implications for medical education

It is understandable that most participants had strongly-held personal beliefs which kept them firmly rooted in the summative assessment culture. For both clinicians and students, it is likely that the prevailing assessment culture throughout school, university and their professional life had been based on high-stakes assessments, with little or no experience of an assessment for learning culture.

If institutions wish to change the assessment and feedback culture within their organisation, it will clearly be insufficient simply to attempt to implement assessment for learning approaches when, despite the evidence of the potential benefits, many stakeholders will be perplexed or resistant to change. Because of cultural conventions of politeness, faculty, clinicians and students may be reluctant to openly express their concerns or beliefs. Indeed, many of the beliefs may be so ingrained and taken for granted within the organisation that they may not be explicitly aware that their personal epistemologies would be an impediment for change.

In order to bring about a change in assessment culture towards one based on programmatic assessment or assessment for learning, the vital factor would appear to be a change in how both students and faculty conceptualise assessment. To accept change, they would need to stop believing in the primacy of summative high-stakes assessments. This requires a radical change in belief and the challenge of how to bring this about should not be underestimated.

When considering such a fundamental change in belief, perhaps we could learn from the field of science education. School science teachers are required to change children’s firmly held preconceptions that the world is flat to an understanding that the earth is spherical. Simply presenting pupils with the apparently irrefutable evidence of a globe fails to convince students of the need to change their beliefs [34]. Instead, Vosniadou [35] argues for what she calls a synthetic models approach to conceptual change within science education. This approach argues that learners form pre-conceptions at an early stage, based on their experience of life. A number of pre-conceptions typically combine together to form a coherent if narrow explanatory framework, sometimes called naïve theory. Vosniadou [35] recognises that conceptual change is not a sudden replacement of one concept with another. Instead it is a slow process that involves a large network of inter-related concepts and which requires the development of new constructions that involve radical changes in personal beliefs. There is a risk with conceptual change that new conceptions are only partially understood, so that pre-conceptions are replaced by misconceptions. Vosniadou argues for beliefs to be challenged so that they are not seen as undisputed scientific facts, but instead as hypotheses to be tested.

Although untested in medical education, Vosniadou’s model appears a plausible proposal for bringing about the huge conceptual change necessary to support the introduction of programmatic assessment. Institutions enthusiastic to implement a change in assessment culture would need to understand the inter-related preconceptions maintaining belief in the summative assessment paradigm. Such preconceptions include the belief that exams are necessary to make students learn, the belief that summative assessments reassure the public that potentially unsafe doctors are prevented from practice and the belief that marking, numbers and grades are more important than feedback.

Although the difficulty of bringing about huge changes in conceptual thinking around assessment may make for somewhat depressing reading, it should be remembered that radical educational innovations can be successful. While the introduction of problem-based learning into medical undergraduate curricula has often been challenging, there are examples of very successful implementation [36]. In Québec, the enactment of change appeared to work as the faculty leaders built on the organisation’s shared beliefs about the faults with the existing curriculum. This was linked to a gradual exposure (over several years) to educational changes occurring elsewhere, which meant that the institution’s own proposed changes did not seem unduly radical [36]. Our study has demonstrated that stakeholders share common beliefs about problems with the current assessment culture, especially with regard to a lack of authenticity in clinical assessments, and there is therefore scope to explore these concerns further as one way of enhancing motivation to change.

There may also be lessons to learn from clinical settings, where there are daily struggles to encourage patients to change unhealthy lifestyles which are contributing to ill health. From the developing literature on health behaviour change, it is clear that listening carefully to patients, and taking time to explore their health beliefs in a non-judgemental manner, can help patients to resolve their own ambivalence about change [37]. Various health behaviour change theories have been used in a range of educational contexts to help researchers understand human behaviour in these settings, with promising results [38]. Our study has demonstrated the insights that can be gained when time is taken to carefully explore stakeholders’ beliefs about assessment culture and their attitude to change.

### Limitations

There are a number of limitations with our study. Studies using interviews and focus groups are inherently limited to considering only the participants’ perspectives. It was conducted in a single institution and it is likely that participants from other sites would have expressed different beliefs. If the group had met on a number of occasions, interactions between stakeholders may well have changed over time. We do not claim to have achieved data saturation. However, the context in which the study took place is not unusual, as most medical schools conduct summative assessment. Indeed, the medical school’s record of innovation in the delivery of feedback may mean that our participants were more open to the possibility of change than might be the case elsewhere. Our findings also appear to resonate with the existing literature on the difficulty of bringing about organisational change [11–13].

### Suggestions for further research

In this study we have explored personal and collective beliefs which would potentially hinder the implementation of a change in assessment culture. Further research is needed to investigate if these findings are replicated in other settings. More work is also required to understand how firmly-held beliefs about summative assessment may be challenged and modified.

## Conclusion

This study has sought to understand the personal and collective beliefs which influence potential redesign of an assessment culture towards one which emphasises assessment for learning. We have shown that a variety of stakeholders hold common assumptions about the primacy of summative assessment. A lack of prior experience of alternative assessment cultures hinders the conceptualisation of radical change. In order to successfully implement a change in assessment culture, firmly-held intuitive beliefs about summative assessment will need to be challenged as a first step.

## Abbreviation

OSCE: Objective structured clinical examination
